# Effect of 5-HT2A Receptor Polymorphisms, Work Stressors, and Social Support on Job Strain among Petroleum Workers in Xinjiang, China

**DOI:** 10.3390/ijerph13121258

**Published:** 2016-12-19

**Authors:** Yu Jiang, Jinhua Tang, Rong Li, Junling Zhao, Zhixin Song, Hua Ge, Yulong Lian, Jiwen Liu

**Affiliations:** Department of Occupational and Environmental Health, College of Public Health, Xinjiang Medical University, Urumqi 830011, Xinjiang, China; jiangyu@stu.xjmu.edu.cn (Y.J.); tang_tg2016@sina.com (J.T.); lurong_128@126.com (R.L.); zhjl8899@sina.com (J.Z.); inforsong@163.com (Z.S.); gehua2710@sina.com (H.G.); lianyulong444@163.com (Y.L.)

**Keywords:** job strain, work stressors, social support, 5-HTR2A, polymorphism

## Abstract

Previous studies have shown that work stressors and social support influence job strain. However, few studies have examined the impact of individual differences on job strain. In Xinjiang, there are a large number of petroleum workers in arid deserts. The present study investigated the effects of work stressors, social support, and 5-hydroxytryptamine receptor (5-HTR2A) genotype on the etiology of job strain among petroleum workers in Xinjiang. A cross-sectional study was carried out between January and August 2013. A total of 700 workers were selected by a three-stage stratified sampling method. 5-HTR2A genotypes were determined with the SNaPshot single nucleotide polymorphism assay. Work stressors and job strain were evaluated with the Occupational Stress Inventory-Revised questionnaire. Social support was assessed with the Chinese Social Support Rating Scale. Work overload and responsibility were significantly associated with job strain. Low social support was associated with severe vocational and interpersonal strain. High social support was a protective factor against job strain (odds ratio (OR) = 0.32, 95% confidence interval (CI): 0.14–0.76). The CC genotype of rs6313 and the AA genotype of rs2070040 were linked to severe vocational strain. Ordinal logistic regression analysis revealed that the CC genotype of rs6313 was linked to higher risk of job strain than the TT genotype (OR = 1.88, 95% CI: 1.10–3.23). These data provide evidence that work stressors, low social support, and 5-HTR2A gene polymorphism contributes to the risk of job strain.

## 1. Introduction

Investigating job-related strain involves studying the relationship between stressful aspects of jobs (i.e., stressor) and the results of exposure to the stressor (i.e., strain). Within this framework, the job strain model [1] proposes that workers exposed to a combination of high job demands and low decision latitude have an increased risk of job strain, which is associated with cardiovascular diseases [2], type 2 diabetes [3], and depression [4]. Stressful experiences at work are often attributed to adverse psychosocial environments. A number of studies have identified work-related stressors that contribute to job strain levels, including poor work environment, professional role ambiguity, tense interpersonal relations, lack of autonomy, and higher work intensity [5,6,7]. One study of healthy workers found that workload was considered a job stressor, reflecting the demands placed upon an employee in their job that cause psychological and behavioral strain [8]. Karasek and Theorell [9] reported a clear association between job strain and levels of social support. The expanded model demonstrated that the support of co-workers and supervisors (workplace support) was one of the most important factors alleviating stress in the work environment. Some reports have shown that help from supervisors and coworkers can prevent occupational strain [10]. In addition to workplace support, social support from family members and close friends can also decrease the effects of job strain [11].

In addition to the aforementioned work-related psychosocial factors, individual differences also dictate the levels personal strain in the workplace [12]. Some research has shown that individuals exhibit a broad range of behavioral and physiological responses to stressors [13]. Adrenal responses to stress—reflecting deviations from normal physiological or psychological set points—demonstrate are a major function of the hypothalamic-pituitary-adrenal (HPA) axis in general adaptation syndrome [14]. This axis is one of the body’s primary hormonal systems for responding to stress and the first to respond to social stressors [15]. Indeed, the basal activity and reactivity of the HPA axis is modulated by and in turn modulates neurotransmitters such as serotonin (5-hydroxytryptamine, 5-HT) [16]. Genetics can predict 5-HT system activation, given its relationship to strain [17]. 5-HTR2A is the main receptor mediating 5-HT stimulation of the HPA axis [18]; the rs6313 single nucleotide polymorphism in the 5-HTR2A gene has been linked to stress-related disorders such as major depression, anxiety, and mood disorder [19,20]. Furthermore, HTR2A variants have been implicated in the neurobiology of posttraumatic stress disorder [21]. However, there is no information available regarding the role of the 5-HT2A receptor gene in job strain.

Employees in the Xinjiang Field Petroleum Administration Bureau have long working hours and arduous tasks, and engage in shift work every 6 months. Their workplaces are installations in arid deserts where they are subjected to isolation, extreme weather, and risk of accidents. Several studies reported that physical and mental health are affected by natural conditions, including the monotonous lifestyle of arid deserts and other harsh environments [22,23]. A positive association between stressors, social support, and outcome (i.e., strain) in employees has been demonstrated [11,24]. Most research has focused on white-collar workers [25,26]; there have been few studies on the relationship between work stressors, social support, 5-HTR2A polymorphism, and job strain in arid deserts petroleum workers. We carried out a cross-sectional study among petroleum workers in Xinjiang to investigate (1) the relationship between work stressors and job strain; (2) the association between social support and job strain; and (3) the relationship between 5-HTR2A polymorphism and job strain. The results provide insight into factors associated with job strain, and underscore the need to establish policies for protecting the health of arid deserts petroleum workers.

## 2. Methods

### 2.1. Study Subjects

This research was part of the Occupational Health Study of Petroleum Workers in China and was carried out between January and August 2013. The study protocol was approved by the Ethics Committee of Xinjiang Medical University (XJMU#2012005). Written informed consent was obtained from all participants. Study subjects were employees aged 20–60 years of both sexes who worked at the Xinjiang Petroleum Administration Bureau of China National Petroleum Corporation in Karamay City, Xinjiang Province, China. A total of 1181 subjects were recruited using a three-stage stratified sampling method. After applying the exclusion criteria, 810 subjects were eligible. Since some of subjects did not agree to participate in the study, 700 workers were ultimately included. In China, workers are required to undergo professional health examinations on a regular basis under the Industrial Safety and Health Act. During the annual professional health examination, we conducted face-to-face interviews with each participant to fill in the questionnaires. The general characteristics noted for each patient were age (<30, 30–40, 40–50, or >50 years), sex, job tenure (<10, 10–20, or >20 years), marital status (unmarried/married), and income level (<3000, 3000–5000, or >5000 yuan).

### 2.2. Work Stressors

We used a validated version of Occupational Stress Inventory-Revised [27], which provides measures for an integrated theoretical model linking sources of stress in the work environment, psychological strains experienced by individuals as a result of work stressors, and coping resources available to combat the effects of stressors and alleviate strain. The questionnaire included three subscales: Occupational Roles Questionnaire (ORQ), Personal Strain Questionnaire (PSQ), and Personal Resources Questionnaire (PRQ). In this study, the ORQ and PSQ were used to evaluate work-related stressors and job strain, respectively.

The ORQ had six dimensions: Role overload, Role insufficiency, Role ambiguity, Role boundary, Responsibility, and Physical environment. Each of the six scales includes 10 items, with scores ranging from 1 to 5. Higher scores on the ORQ indicated more work stressors.

### 2.3. Job Strain

The Personal Strain Questionnaire (PSQ) measures the outcome of work stressors manifested as personal strain within the following categories: Vocational strain, Psychological strain, Interpersonal strain, and Physical strain. Subjects responded to each item on a five-point Likert scale ranging from 1 (never) to 5 (always). For PSQ subscales, a higher score indicated a severe level of job strain.

We used standard scoring for the PSQ to further classify the job strain group. Raw scores for each dimension were converted to T scores (mean ± SD, 50 ± 10) based on the general population of China [28]. T scores ≥70, between 60 and 69, and ≤59 indicated high, moderate, and low levels of job strain, respectively [28].

### 2.4. Social Support Assessment

Social support was measured with the Chinese Social Support Rating Scale [29], which consists of 10 items divided into three dimensions: objective and subjective support and use of social support. Higher scores indicated better social support from family, friends, and colleagues. Each item was scored differently: maximum scores of 4 for items 1–4 and 8–10, 20 for item 5, and 9 for items 6 and 7, for a total score of 66. Total scores <33, 33–45, and <45 indicated low, moderate, and high levels of social support, respectively.

### 2.5. DNA Sampling and Genotyping

Venous blood samples were obtained as part of the health examination and were anonymized. Samples were collected in EDTA-containing tubes from each participant following a 12-h fast. Genomic DNA was purified from the samples using a whole blood genome extraction kit (Tiangen Biotech, Beijing, China) and stored at −20 °C until use. Tag single nucleotide polymorphisms (SNPs) of 5-HTR2A in the Chinese Han population were identified in the Haplotype Map database (National Center for Biotechnology Information, Bethesda, MD, USA). The SNPs rs6313, rs1923884, and rs2070040 were genotyped with the SNaPshot SNP assay. Data were analyzed using GeneMapper 4.1 (Applied Biosystems, Foster City, CA, USA).

### 2.6. Statistical Analysis

Data were analyzed using SPSS for Windows v.17.0 software (SPSS Inc., Chicago, IL, USA). The χ^2^ test was used to analyze the distribution of general characteristics among different job strain groups and evaluate Hardy-Weinberg equilibrium. Hardy-Weinberg equilibrium (HWE) is assumed when observed genotype and allele frequencies between parents and their offspring are in equilibrium in a population. When *p* > 0.05, the research object is representative of the general population. Job strain scores of different genotypes and social support groups were analyzed as continuous variables and evaluated by one-way analysis of variance. Odds ratios (ORs) and 95% confidence intervals (CIs) were determined for risk associated with job strain by ordinal logistic regression. The tests were two-tailed and the significance level was set at *p* < 0.05.

## 3. Results

### 3.1. Characteristics of Subjects and Associations with Job Strain

The characteristics of the 700 study subjects are shown in Table 1. There were no statistically significant associations between job strain and age, job tenure, marital status, or income level (*p* > 0.05).

### 3.2. Association between Work Stressors and Job Strain

There were significant differences in terms of role overload and responsibility among low, moderate, and high job strain groups (*p* < 0.05). These were further examined with the Tukey test for multiple comparisons. For role overload, scores were higher for the high and moderate job strain groups as compared to the low job strain group (*p* < 0.05). For responsibility, scores were higher for the high job strain as compared to the low and moderate job strain groups (*p* < 0.05; Table 2).

### 3.3. Association between Job Strain and Social Support

Ordinal logistic regression analyses were carried out to assess the relationship between job strain and social support (Table 3). High social support was a protective factor against job strain (OR = 0.32, 95% CI: 0.14–0.76).

### 3.4. Social Support Level Is Associated with Job Strain Subscores

We found significant differences in terms of vocational and interpersonal strain among the low, moderate, and high social support groups (*p* < 0.05). These were further examined with the Tukey test for multiple comparisons, which revealed higher scores for the low as compared to the high social support group for both subscales of strain (*p* < 0.05; Table 4).

### 3.5. Association between 5-HTR2A Polymorphism and Job Strain

5-HTR2A genotypes were in Hardy-Weinberg equilibrium and there were no differences in job strain groups (*p* > 0.05; Table 5). For rs6313, a χ^2^ analysis revealed differences between genotype distributions among low, moderate, and high job strain groups (*p* < 0.05). Similarly, ordinal logistic regression analysis showed that the CC allele (OR = 1.88, 95% CI: 1.10–3.23) increased the risk of high job strain as compared to the TT allele (Table 5).

### 3.6. 5-HTR2A Gene Polymorphisms Are Related to Job Strain Subscores

For the rs6313 single nucleotide polymorphism of 5-HTR2A, differences were observed between the three genotype groups in terms of vocational strain (*p* < 0.05). An analysis with the Tukey test for multiple comparisons found higher scores for the CC than for the CT genotype (*p* < 0.05; Table 6). 

For rs1923884, there were no statistically significant associations among the three genotypes in terms of job strain subscales (*p* > 0.05; Table 7). 

For rs2070040, there were significant differences in vocational strain among the three genotypes; the Tukey test for multiple comparisons revealed a higher score for the AA as compared to the AG genotype (*p* < 0.05; Table 8).

## 4. Discussion

This study investigated the relationship between 5-HTR2A gene polymorphisms, work stressors, social support, and job strain among Chinese workers. We found that higher scores for role overload and responsibility were associated with high job strain level, which is consistent with previous findings on occupational strain among Chinese Han teachers [30]. Petroleum workers often work overtime and night shifts, and spend an excessive amount of time and energy completing their performance goals; they therefore lack the time to find relief from the competitive pressures they face. In a Canadian study, responsibility was related to greater depersonalization, feelings of lower personal accomplishment, and greater somatization and strain [31]. The absence of proper monitoring in this line of work can lead to accidents, environmental pollution, or corporate property damage; as a result, oil workers bear more responsibility, which causes a higher level of strain.

Social support plays a significant role in an individual’s ability to cope with job strain. This is consistent with previous findings showing that a higher level of job strain is observed in individuals with lower levels of support [32,33]. Moreover, it has been reported that women with low social support from their colleagues had higher levels of cortisol, the hormone released during strain [34]. Several studies have shown that interpersonal relationships with clients, coworkers, and supervisors are important predictors of psychological distress [35]. Interpersonal strain represents a specific disengagement reaction towards demanding interpersonal interactions and social pressures at work, through which an individual creates emotional and cognitive distance from others [27]. We found that low social support increased the risk of experiencing job strain, especially vocational and interpersonal strain.

Few studies to date have investigated the association between 5-HTR2A genes and job strain. The results of our study indicate that the AA genotype of rs2070040 and CC genotype of rs6313 were associated with serious vocational strain. The observed associations between the three single nucleotide polymorphisms and job strain provide further evidence of the functional role of the 5-HTR2A gene in the development of job strain. Some studies have found a link between the A allele of rs2070040—an SNP in the 5-HTR2A promoter—and postpartum depression [36]. In addition, compared to the T variant, the C allele of rs6313 was more closely associated with lower 5-HTR2A mRNA and protein expression [37], which may be a response to increased levels or release of 5-HT in the brain. Moreover, 5-HT regulates the release of the HPA axis peptides corticotropin-releasing hormone and adrenocorticotropic hormone, along with the adrenal hormone cortisol [38] to modulate the stress response. 5-HT2A receptors of the hypothalamic paraventricular nucleus (PVN) participate in HPA axis activation [39]. The high-risk CC genotype has been linked to various stress-related disorders [40,41].

There were some limitations to the present study. Firstly, due to its cross-sectional nature, the results suggested an association between 5-HTR2A gene polymorphism and job strain but did not indicate a causal relationship. Secondly, we used a self-reported questionnaire to measure job strain levels; the responses may have therefore lacked accuracy and yielded false-positive results. Ideally, a quantitative analysis of serum cortisol levels should be carried out to substantiate the results of the questionnaire. Thirdly, the study subjects were all from the Chinese Han population, and therefore the results may not be generalizable to other ethnic groups and populations, which would require a longitudinal cohort study. Finally, we investigated only three tag SNPs, but it is possible that other polymorphisms of the 5-HTR2A gene affect susceptibility to job strain.

## 5. Conclusions

The Occupational Stress Inventory-Revised Questionnaire, Chinese Social Support Rating Scale, and SNaPshot single nucleotide polymorphism assay were used to evaluate the relationship between work stressors, social support, and 5-HT2A receptor polymorphisms on job strain in petroleum workers in arid deserts. The results revealed that work overload and responsibility were strongly associated with job strain in petroleum workers, and that low social support and the CC genotype of the 5-HTR2A gene were risk factors for job strain. Our findings demonstrate the effects of genetics and environmental factors on the etiology of job strain, and suggest possible factors that account for differences in individual susceptibility. Based on these results, our primary policy suggestion is to set up a proactive workplace intervention to reduce job strain in petroleum workers (focusing on carriers of the CC genotype), including measures to promote good interpersonal relationships with co-workers, supervisors, family, and friends and minimize the risk of work stressors.

## Figures and Tables

**Table 1:** Job strain groups according to subject characteristics.

Characteristics	N	Job Strain Level			χ^2^	*p* Value
		High	Moderate	Low		
Age (years)						
<30	152	32	52	68	7.006	0.320
30–40	303	82	102	119		
40–50	230	68	84	78		
>50	15	3	4	8		
Sex						
Male	303	70	113	120	3.432	0.180
Female	397	115	129	153		
Job tenure (years)						
<10	221	54	75	92	1.511	0.825
10–20	196	51	68	77		
>20	283	80	99	104		
Marital status						
Not married	98	19	37	42	2.906	0.234
Married	602	166	205	231		
Income level (yuan)						
<3000	105	23	42	40	3.882	0.422
3000–5000	565	157	188	220		
>5000	30	5	12	13		

**Table 2:** Association between work stressors and job strain level.

Work Stressors	Job Strain Level			*F*	*p*
	Low	Moderate	High		
Role overload	27.46 ± 6.90	28.87 ± 6.74	30.01 ± 7.98	7.372	0.001
Role insufficiency	31.58 ± 7.01	32.05 ± 7.55	30.86 ± 7.37	0.439	0.645
Role ambiguity	32.98 ± 7.51	33.31 ± 7.14	32.60 ± 7.47	0.375	0.687
Role boundary	28.83 ± 6.37	29.79 ± 7.06	30.22 ± 7.14	0.139	0.870
Responsibility	27.02 ± 6.78	28.47 ± 7.36	29.03 ± 7.67	4.228	0.021
Physical environment	30.23 ± 7.03	31.63 ± 7.15	31.29 ± 7.50	1.593	0.204

**Table 3:** Association between job strain and social support.

Social Support	Job Stain Level			OR (95% CI)	*p* Value for OR
	High	Moderate	Low		
Low	62	83	96	Reference	-
Moderate	120	145	156	0.49 (0.21–1.13)	0.095
High	3	14	21	0.32 (0.14–0.76)	0.010 *

CI, confidence interval; OR, odds ratio. * *p* < 0.05.

**Table 4:** Association between social support level and job strain subscales.

Job Strain	Social Support Level			*F*	*p*
	Low	Moderate	High		
Vocational strain	30.02 ± 7.19	28.75 ± 6.43	27.36 ± 6.46	3.452	0.032
Psychological strain	29.48 ± 6.27	30.70 ± 7.66	29.56 ± 6.55	2.821	0.060
Interpersonal strain	30.75 ± 5.36	30.34 ± 5.44	29.65 ± 5.48	3.302	0.037
Physical strain	29.03 ± 6.08	29.23 ± 6.86	28.53 ± 6.75	2.256	0.105

**Table 5:** Association between 5-HTR2A polymorphism and job strain.

Genotype	N	Job Strain Level			OR (95% CI)	*p* Value for OR
		High	Moderate	Low		
rs6313						
TT	125	24	39	62	1.00	-
CT	334	87	121	126	1.35 (0.78–2.36)	0.284
CC	241	74	82	85	1.88 (1.10–3.23)	0.021 *
*p*-value for χ^2^				0.049		
*p*-value for HWE		0.842	0.612	0.245		
rs1923884						
CC	178	51	58	69	1.00	-
CT	346	90	122	134	0.70 (0.44–1.11)	0.129
TT	176	44	62	70	0.81 (0.47–1.38)	0.436
P2				0.805		
P3		0.727	0.894	0.762		
rs2070040						
AA	120	38	50	32	1.00	-
AG	329	83	115	131	1.43 (0.94–2.19)	0.095
GG	251	64	77	110	1.53 (0.90–2.59)	0.119
P2				0.334		
P3		0.250	0.559	0.457		

CI, confidence interval; HWE, Hardy-Weinberg equilibrium; OR, odds ratio. * *p* < 0.05.

**Table 6:** Association between rs6313 polymorphism and job strain subscales.

Job Strain	rs6313			*F*	*p*
	CC	CT	TT		
Vocational strain	26.76 ± 6.87	25.31 ± 6.44	25.53 ± 5.93	3.126	0.042
Psychological strain	27.58 ± 7.29	26.68 ± 7.09	26.89 ± 7.20	2.536	0.080
Interpersonal strain	28.22 ± 5.95	27.41 ± 5.95	27.15 ± 6.69	0.707	0.494
Physical strain	27.21 ± 6.03	27.01 ± 6.16	28.07 ± 6.63	0.407	0.666

**Table 7:** Association between rs1923884 polymorphism and job strain subscales.

Job Strain	rs1923884			*F*	*p*
	CC	CT	TT		
Vocational strain	25.75 ± 6.64	25.92 ± 6.50	25.81 ± 6.50	0.156	0.856
Psychological strain	27.66 ± 7.63	26.68 ± 7.01	27.06 ± 7.04	0.625	0.536
Interpersonal strain	27.80 ± 6.18	27.69 ± 6.11	27.39 ± 5.99	1.100	0.333
Physical strain	27.39 ± 6.37	27.18 ± 6.37	27.37 ± 5.72	0.054	0.947

**Table 8:** Association between rs2070040 polymorphism and job strain subscales.

Job Strain	rs2070040			*F*	*p*
	AA	AG	GG		
Vocational strain	26.41 ± 6.86	25.08 ± 6.19	25.93 ± 6.17	3.437	0.034
Psychological strain	26.98 ± 7.31	27.56 ± 6.97	26.35 ± 7.36	0.798	0.451
Interpersonal strain	27.87 ± 5.62	27.99 ± 6.13	27.08 ± 6.23	0.937	0.392
Physical strain	27.18 ± 5.82	27.39 ± 6.32	27.16 ± 6.24	0.043	0.958
